# Universum Inspired Graph Contrast Learning for Major Depressive Disorder Identification: In Functional Brain Network View

**DOI:** 10.1002/cns.71158

**Published:** 2026-09-21

**Authors:** Hao Qin, Guanghui Hong, Di Ma, Zeyi Wang, Leina Zhao, Weikai Li, Xiaowen Xu

**Affiliations:** ^1^ College of Information Science and Engineering Chongqing Jiaotong University Chongqing China; ^2^ Department of Radiology and Nuclear Medicine, School of Medicine, Tongji Hospital Tongji University Shanghai China; ^3^ Institute of Medical Imaging Artificial Intelligence Tongji University School of Medicine Shanghai China; ^4^ Department of Neurosurgery The First Affiliated Hospital of Fujian Medical University Fuzhou China; ^5^ College of Information Science and Technology & Artificial Intelligence Nanjing Forestry University Nanjing China; ^6^ School of Mathematics and Statistics Chongqing Jiaotong University Chongqing China; ^7^ Key Laboratory of Complex Systems Optimization and Intelligent Control of Chongqing Municipal Education Commission Chongqing Jiaotong University Chongqing China; ^8^ School of Computer and Artificial Intelligence Shandong Jianzhu University Jinan China

**Keywords:** clinical interpretability, contrast learning, functional brain network, functional magnetic resonance imaging, graph convolutional network, major depressive disorder

## Abstract

**Background:**

Major depressive disorder (MDD) is a serious psychological disorder marked by persistent feelings of sadness and a notable decline in daily functioning. Functional Brain Networks (FBNs) have become crucial biomarkers for the diagnosis of MDD, and Graph Convolutional Networks (GCNs) have demonstrated great potential in extracting FBN features. However, existing GCN methods typically depend on large‐scale labeled datasets and lack clinical interpretability in MDD identification, which limits their application.

**Methods:**

We introduce Universum Inspired Graph Contrast Learning (Uni‐GCL), a novel graph‐based self‐supervised learning framework to relieve the demand for training sample size. Additionally, to enhance the clinical interpretability of GCNs, we optimized the Gradient‐weighted Class Activation Mapping (Grad‐CAM) method to enable it to effectively pinpoint the crucial nodes within the GCN.

**Results:**

The Uni‐GCL method outperformed previous state‐of‐the‐art approaches. It improved accuracy by 3% at Site 21 and 5% at Site 1. Additionally, Uni‐GCL identified that brain regions FGoperc. R, CAU. L, FFG. R, AMYG. L, PUT. L, AMYG. R, PUT. R, THA. L, IFGtriang. R, FFG. L, INS. L, INS. R, HIP. L and HIP. R make significant contributions to the diagnosis of MDD.

**Conclusions:**

Uni‐GCL can effectively reduce the sample size for model training and improve the diagnostic performance of MDD. Meanwhile, the adapted Grad‐CAM provides an effective tool to enhance the interpretability of GCN.

## Graphical abstract

Uni‐GCL pretrains a spatio‐temporal GCN on rs‐fMRI functional brain networks using two‐level augmentation and mixup‐based universum samples for self‐supervised contrastive learning. With only a small labeled target set, it improves cross‐site MDD identification and provides node‐wise interpretability via graph Grad‐CAM.

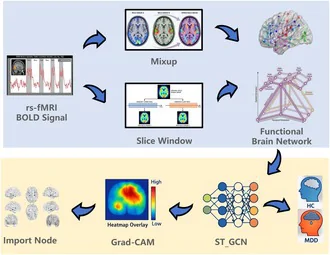

## 1 Introduction

Major depressive disorder (MDD) [1] is a common emotional disorder that causes persistent low mood, a lack of pleasure, and may lead to a range of physiological, psychological, social, and personality disorders [2, 3, 4]. The current mainstream diagnostic system for MDD mainly relies on symptom‐based clinical interviews and criteria such as DSM‐5 [5], while neuroimaging examinations (e.g., structural MRI) are generally used as auxiliary tools to rule out organic brain abnormalities. However, there are significant deficiencies in practical applications. Clinical evaluation relies on the subjective judgment of psychiatrists based on structured interview scales such as SCID (Structured Clinical Interview for DSM‐5), and self‐reports are susceptible to recall bias and covert responses. Thus, developing more effective methods for early identification and revealing the biomarkers of MDD are imperative.

Currently, the development of machine learning has provided a new direction for MDD diagnosis techniques [6, 7]. For instance, several MDD diagnosis studies combine functional brain networks (FBNs) with machine learning techniques [8], such as Support Vector Machines (SVM), Random Forests (RF) [9, 10], and Graph Kernels [11, 12]. Nevertheless, these methods may not fully capture the topological information between brain regions and their relationship to brain saliency patterns when extracting static statistical features. Here, we use the term brain saliency patterns to refer to salient disease‐related patterns of functional connectivity and neural activity that make prominent contributions to distinguishing MDD patients from healthy controls. Inspired by the great potential of Graph Convolutional Networks (GCNs) in machine learning, the GCN‐based model has come to the mainstream in disease diagnosis [13, 14, 15, 16, 17, 18]. However, these GCN‐based methods typically require a large amount of annotated data, which is often difficult to obtain in the medical domain [19]. Moreover, although GCN‐based models have shown promising performance, their decision process remains largely opaque from a clinical perspective and lacks clinical interpretability, which limits their direct translational utility in MDD diagnosis.

**FIGURE 1:**
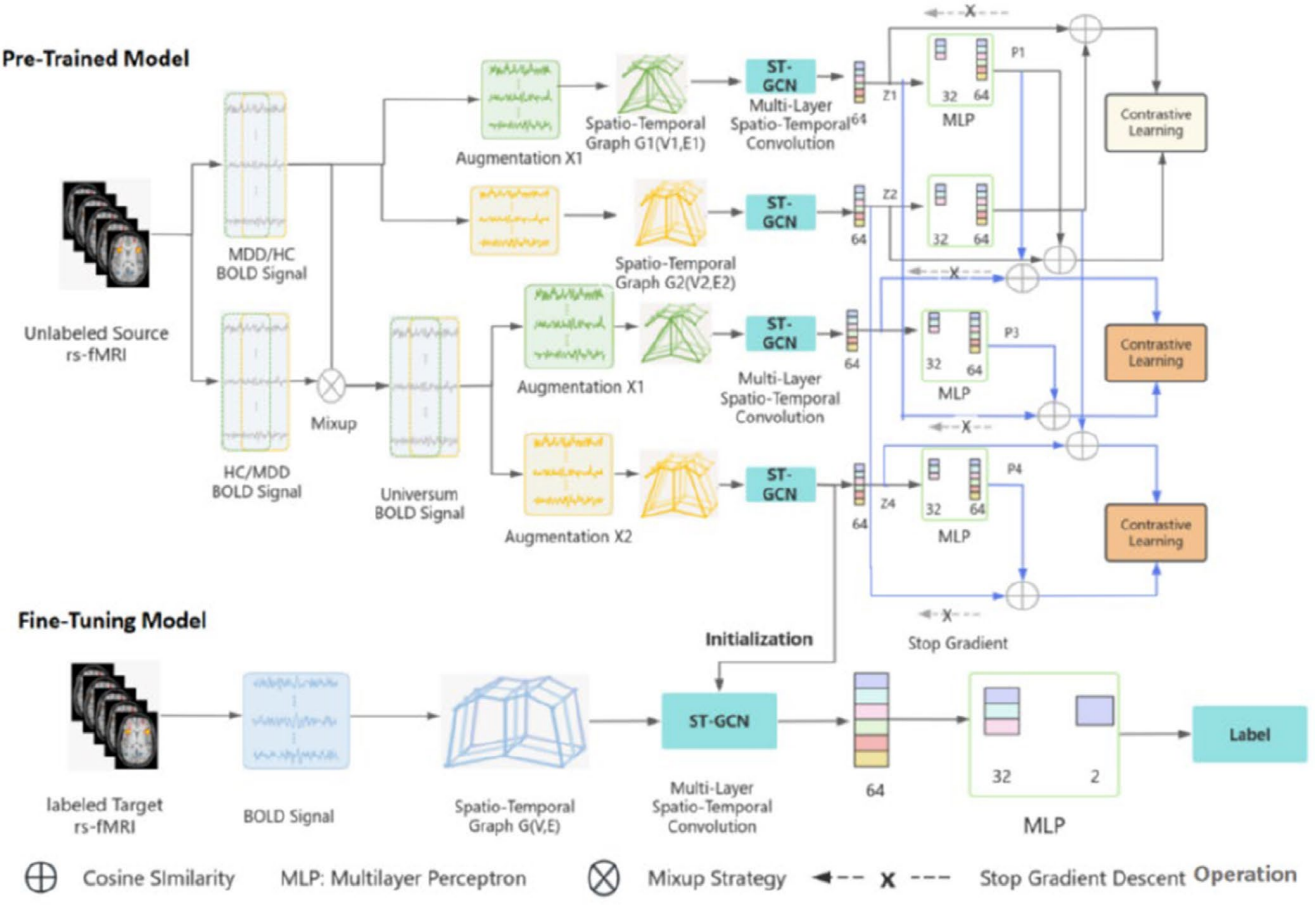
Illustration of the proposed universum Inspired Graph Contrastive Learning framework (Uni‐GCL), consisting of a pre‐trained model and a fine‐tuning model.

Compared with conventional supervised contrastive learning, self‐supervised methods generate supervised signals in the pre‐training stage by designing data‐driven pretext tasks, rather than relying on manually annotated labels [20]. To release the dilemma of the data requirements, we established a self‐supervised learning framework called Universum Inspired Graph Contrastive Learning (Uni‐GCL), as shown in Figure 1. In particular, the Uni‐CGL consists of two parts: (1) a pre‐trained model trained on unlabeled data, and (2) a fine‐tuning model for analyzing the target data. Specifically, in the pre‐trained model, we construct universum samples through the mixup strategy to introduce more abundant discriminative information. This design provides additional “inter‐class constraints” for contrastive learning, enhancing the learned ability to distinguish representations [21, 22]. Furthermore, a two‐level fMRI enhancement strategy is utilized to increase the sample size by enhancing the blood oxygen level dependent (BOLD) signal for both the original and mixed samples and using a parallel spatio‐temporal graph convolutional network (ST‐GCN) for fMRI feature extraction. Additionally, to fully utilize these universum samples, we designed a contrastive loss function to handle the differences between positive samples and universum samples [23, 24]. Then, in the fine‐tuning phase, we only need to utilize a small amount of annotated data to further fine‐tune the model. To validate the performance of the proposed Uni‐CGL method, we conducted cross‐site learning tasks on the REST‐MDD dataset. Moreover, to enhance the interpretability of our Uni‐GCL, we adopt Gradient‐weighted Class Activation Mapping (Grad‐CAM) [25], a widely used post hoc gradient‐based visualization method that uses the gradients of a target class with respect to the final feature maps to produce class‐discriminative localization maps. In our setting, we extend Grad‐CAM from convolutional neural networks to graph convolutional networks (GCNs) on functional brain networks, in order to highlight the brain regions (nodes/ROIs) that most strongly support the MDD diagnosis. It provided interpretable links between learned representations and established neurobiological mechanisms of depression. The main contributions of this paper are summarized as follows:

- We introduce the Universum learning paradigm to MDD neuroimaging classification tasks. By leveraging Universum samples, we effectively alleviate the long‐standing bottleneck of labeled data scarcity and introduce additional inter‐class constraints into the contrastive learning process, thereby significantly enhancing the model's generalization ability under limited labeled data conditions.
- We adapt the Grad‐CAM technique to ST‐GCN architectures for functional brain network analysis. While Grad‐CAM has been widely adopted in the field of computer vision, its application to ST‐GCN structures in neuroimaging analysis remains relatively understudied. Our improved method can accurately identify key brain regions associated with depression, establishing interpretable links between model predictions and the neurobiological mechanisms of depression.

## 2 Materials and Methods

### 2.1 Demographic and Clinical Characteristics

This study utilizes data from the REST‐meta‐MDD dataset. To minimize selection bias and ensure the validity of experimental outcomes, we select 442 MDDs and 395 HCs from the available subjects. At each site, patient and healthy control samples are balanced at a nearly 1:1 ratio; detailed demographic and clinical information is provided in Table 1, and efforts are made to match gender composition and maintain comparable age distributions between groups.

**TABLE 1:** Demographic and clinical information of subjects from REST‐MDD datasets.

Site ID/name	Category	Scans	Gender (M/F)	Age (mean ± SD)
1	MDD	74	31/43	31.72 ± 9.19
1	HC	74	32/42	31.80 ± 8.99
20	MDD	282	99/183	38.74 ± 13.74
20	HC	251	87/164	39.64 ± 15.87
21	MDD	86	38/48	34.71 ± 12.63
21	HC	70	31/39	36.13 ± 12.64

In our cross‐site evaluation protocol, Uni‐GCL is trained exclusively on Site 20, using 282 MDDs and 251 HCs (533 subjects in total) as the training set. The trained model is then independently tested on Site 1 with 74 MDDs and 74 HCs (148 subjects) and on Site 21 with 86 MDDs and 70 HCs (156 subjects), without any subject overlap between training and testing. This standardized setup, which follows previous REST‐meta‐MDD based studies, ensures a transparent separation between training and testing data and mitigates confounding effects associated with site‐specific characteristics. Furthermore, we also conducted cross‐data experiments on the ADNI dataset, as detailed in Table A1.

### 2.2 MRI Acquisition and Preprocessing

REST‐MDD [26] rs‐fMRI data were preprocessed via the Data Processing Assistant for Resting‐State fMRI (DPARSF) pipeline [27]. Specifically, the first 10 volumes are first discarded to obtain a stable magnetization state, followed by slice timing correction, head motion correction, bandpass filtering (0.01–0.1 Hz), regression of nuisance covariates (i.e., head motion parameters, white matter and cerebrospinal fluid) [28]. Then, the coregistration between T1 weighted and functional images is performed using a 6 degrees‐of‐freedom linear transformation and unified segmentation‐based normalization was then applied to warp the functional images from native space into the MNI standard space with 3 × 3 × 3 mm^3^ isotropic voxels. Finally, each brain is partitioned into 116 ROIs based on the Automated Anatomical Labeling (AAL) atlas [29] and the average time series are extracted.

To validate the generalization performance of our method, we utilized multi‐site MDD data with heterogeneous scanning parameters for experimental verification. For Site 20, resting‐state functional MRI (rs‐fMRI) data were acquired using a Siemens Tim Trio 3T scanner equipped with a 12‐channel receiver coil. The scanning parameters for each fMRI were as follows: repetition time (TR) = 2000 ms, echo time (TE) = 30 ms, voxel size = 3.44 × 3.44 × 4.00 mm^3^, slice number = 32, slice thickness = 3.0 mm, gap = 1.0 mm, number of time points = 242, field‐of‐view (FOV) = 220 × 220 mm^2^, and flip angle = 90°.

For Site 21, data were acquired using a Siemens Tim Trio 3T scanner with a 32‐channel receiver coil. The scanning parameters for each fMRI were as follows: TR = 2000 ms, TE = 30 ms, voxel size = 3.12 × 3.12 × 4.20 mm^3^, slice number = 33, slice thickness = 3.5 mm, gap = 0.7 mm, number of time points = 240, FOV = 200 × 200 mm^2^, and flip angle = 90°.

Additionally, the rs‐fMRI data were acquired using a Siemens Tim Trio 3T scanner equipped with a 32‐channel receiver coil in Site 1. The scanning parameters for each fMRI were as follows: TR = 2000 ms, TE = 30 ms, voxel size = 3.28 × 3.28 × 4.80 mm^3^, slice number = 30, slice thickness = 4.0 mm (noted as 40 mm in the original text, likely a typo), gap = 0.8 mm, number of time points = 210, FOV = 210 × 210 mm^2^, and flip angle = 90°.

### 2.3 Universum Inspired Graph Contrast Learning

To alleviate the reliance on samples and improve the model performance, we specially designed a Universum Inspired Graph Contrastive Learning method (Uni‐GCL), which mainly includes the following parts, as detailed in Figure 1.

### 2.4 Mixup Based Universum Samples

We denote each subject as $X_{i}\in R^{T\times V}$, where T is the number of time points after discarding the initial volumes and V=116 is the number of ROIs defined by the AAL atlas. Each column of $X_{i}$ corresponds to the fMRI time series of one ROI. Before constructing functional connectivity, the raw time series are directly used to compute Pearson correlation coefficients, which are inherently scale‐invariant. To generate universum samples, we apply a mixup operation between samples from different classes [30]:

(1) $${\hat{X}}_{i}=\lambda X_{i}+\left(1-\lambda \right)X_{j},$$

where $X_{i}$, $X_{j}\in R^{T\times V}\left(i,j=1,\ldots ,N\right)$ are the normalized samples selected from different groups, N represents the total number of samples therein, λ=0.5 in this study, and ${\hat{X}}_{i}\in R^{T\times V}$ denotes the resulting universum sample. For each sample in the training set, a sample from a different class is randomly selected to construct ${\hat{X}}_{i}$.

For each (original or universum) sample, we compute an adjacency matrix $A\in R^{V\times V}$, where $A_{\mathrm{pq}}=|\text{corr}\left(X_{:,p},X_{:,q}\right)|$ denotes the absolute Pearson correlation between ROI p and ROI q. We then apply symmetric normalization $\tilde{A}=D^{-1/2}AD^{-1/2}$, where D is the degree matrix of A. This normalization stabilizes graph convolution and emphasizes the relative structural relationships between nodes.

### 2.5 Contrastive Learning

Then, these synthetic samples are integrated as negative examples within the contrastive loss framework during model training. Specifically, to make use of universum samples and perform model pre‐training, we specially designed a contrastive loss as follows:

(2) $$\begin{array}{c} \begin{array}{c} \xi =\frac{1}{2}(\psi (g(z_{1}),p_{2})+\psi (g(z_{2}),p_{1})-\frac{1}{4}(\psi (g\left(z_{1}\right),{\hat{p}}_{1}\\ +\psi (g\left({\hat{z}}_{1}\right)),p_{1})+\psi (g(z_{2}),{\hat{p}}_{2})+\psi (g({\hat{z}}_{2}),p_{2})), \end{array} \end{array}$$

From Equation (2), $z_{i}$ and ${\hat{z}}_{i}\left(i=1,2\right)$ denote the feature representations of the original samples and universum samples after data augmentation across two views, respectively. $p_{i}$ and ${\hat{p}}_{i}\left(i=1,2\right)$ denote the output of $z_{i}$ and ${\hat{z}}_{i}$ processed by a Multi‐layer Perceptron (MLP). $\psi \left(\cdot \right)$ represents negative cosine similarity and $g\left(\cdot \right)$ represents the gradient stopping operation.

### 2.6 Two‐Level fMRI Augmentation and Feature Extraction

**FIGURE A1:**
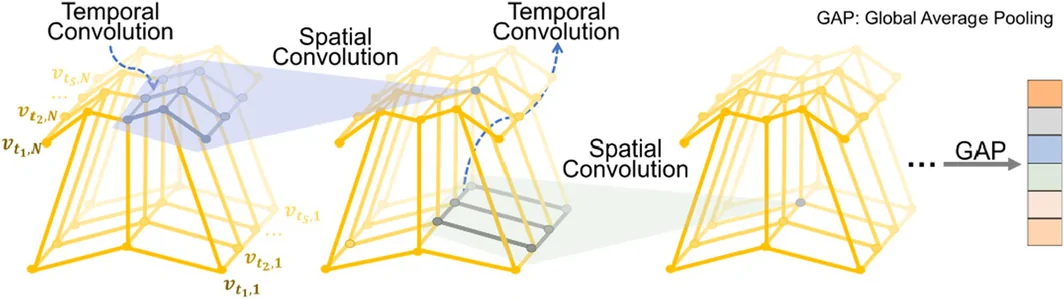
Details of convolution operations in ST‐GCN feature extractor.

Finally, for pre‐training the model, we introduced a data augmentation method that utilizes BOLD signals, which improves the generalization performance of deep learning models in fMRI data analysis and alleviates overfitting. This method employs a window‐slicing strategy to prune the initial 90% and final 90% segments of the original time series to generate new samples [31], thereby expanding the original BOLD time series. And it preserves functional connectivity information and prevents direct enhancement of 4D functional magnetic resonance scanning. Importantly, this method effectively extends time series data without introducing extra noise or altering temporal dependencies [32]. After data augmentation, to enhance the generalization ability of the model in fMRI data analysis, we employed a self‐supervised contrastive learning framework; the details can be found in the Appendix. The detailed feature extraction process of ST‐GCN is shown in Figure A1.

### 2.7 Model Fine‐Tuning

The refined architecture consists of an ST‐GCN feature extractor, two dense layers within an MLP, and a SoftMax prediction layer. During supervised training, the model utilizes labeled target fMRI data to generate spatiotemporal maps and extract discriminative features via the ST‐GCN module. Brain disease classification is subsequently achieved by the MLP and SoftMax layers, with the entire framework optimized through minimizing cross‐entropy loss.

### 2.8 Grad‐CAM for Node‐Wise Importance

Subsequently, to visualize and analyze which brain regions drive the decisions of our GCN‐based classifier, we adapt Gradient‐weighted Class Activation Mapping (Grad‐CAM) to graph‐structured fMRI data and compute node‐wise importance scores. Different from directly inspecting the non‐linear activation function, our maps are derived from the pre‐softmax logits and quantify a conditional sensitivity: for each ROI, we measure how the class score changes under small perturbations of its representation while keeping all other regions fixed rather than assuming a univariate effect of a single region.

Let $f\left(\cdot ;\theta \right)$ be the classifier and $z\left(x\right)=f\left(x;\theta \right)\in R^{C}$ the logits for subject x. Denote by $H\left(x\right)\in R^{T\times N\times K}$ the feature maps at the target layer (time *T*, nodes *N* = 90, channels *K*). Define the scalar score $S\left(x\right)=z_{c}\left(x\right)$, where c is the true label (or predicted class) of sample x. For each subject x, we first compute Grad‐CAM channel weights by averaging the gradients of $S\left(x\right)$ over all time points and nodes:

(3) $$\alpha_{k}\left(x\right)=\frac{1}{\mathrm{TN}}\sum_{t=1}^{T}\sum_{i=1}^{N}\frac{\partial S\left(x\right)}{\partial H_{t,i,k}\left(x\right)},k=1,\ldots ,K,$$

These $\alpha_{k}\left(x\right)$ measure how sensitive the class score is to each channel *k* at the target layer.

We then obtain a node‐wise Class Activation Map (CAM). First, we average the feature maps over time to obtain node‐wise features ${\tilde{H}}_{i,k}\left(x\right)=\frac{1}{T}\sum_{t}H_{t,i,k}\left(x\right)$. Next, we aggregate across channels using the Grad‐CAM weights:

(4) $${\mathrm{CAM}}_{i}\left(x\right)=\text{ReLU}\left(\sum_{k=1}^{K}\alpha_{k}\left(x\right){\tilde{H}}_{i,k}\left(x\right)\right),i=1,\ldots ,N,$$

For each node i, ${\mathrm{CAM}}_{i}\left(x\right)$ is a non‐negative sensitivity value indicating how strongly ROI i contributes to the class score $S\left(x\right)$, conditional on all other ROIs.

For visualization and comparison across subjects, we normalize the node‐wise CAMs of each subject to a fixed range [2, 5]:

(5) $$C\hat{A}M_{i}\left(x\right)=2+3\cdot \frac{{\mathrm{CAM}}_{i}\left(x\right)-\min_{j}{\mathrm{CAM}}_{j}\left(x\right)}{\max_{j}{\mathrm{CAM}}_{j}\left(x\right)-\min_{j}{\mathrm{CAM}}_{j}\left(x\right)+\varepsilon },$$

where ε is a small constant for numerical stability. The values $C\hat{A}M_{i}\left(x\right)$ are used to control node size or color in the brain plots. To obtain site‐level importance, we average the normalized node‐wise CAMs over all subjects $X_{s}$ at site s (s=1,20,21):

(6) $$S_{i}^{\left(s\right)}=\frac{1}{|X_{s}|}\sum_{x\in X_{s}}C\hat{A}M_{i}\left(x\right),i=1,\ldots ,N,$$

Grad‐CAM is computed on the logits $S\left(x\right)$ (pre‐softmax) to avoid gradient saturation. The target layer is chosen as the final graph block before global pooling/fully connected layers that still retains node resolution. The ReLU operation discards negative contributions and focuses on features that positively support the predicted class. All node indices refer to the AAL90 atlas (Algorithm 1). The pseudo‐code is as follows:

ALGORITHM 1 **Graph‐Grad‐CAM node importance estimation (ROI attribution).**

Input: The trained model M, training set $D=\left\{X,Y\right\}$, adjacency matrix A, target layer L(the last layer that still retains “node resolution”), nodes number N

Output: Node importance vector $S\in R^{N}$

for batch_size B∈D do.

$\text{logtis}\leftarrow M\left(x_{B},A\right)$

$c_{B}\leftarrow \text{argmax}\left(\text{logtis},\text{axis}=1\right)$

$\text{score}\leftarrow \sum_{b}\text{logtis}\left(b,c_{B_{b}}\right)$

Save the activation of L as $A_{\text{feat}}$

Save $\partial \;\text{score}/\partial \;A_{\text{feat}}=d\;A_{\text{feat}}$

Back propagation score to obtain $A_{\text{feat}}$ and $d\;A_{\text{feat}}$

$a_{k}\leftarrow \text{mean}\left(d\;A_{\text{feat}}\right)$

${\mathrm{CAM}}_{\text{space}}\leftarrow \text{RELU}\left(\sum_{k}a_{k}⊙A_{\text{feat}}\left[1,2,\ldots ,k\right]\right)$

${\text{cams}}_{B}\leftarrow \text{mean}\left({\mathrm{CAM}}_{\text{space}}\right)$

Normalize each sample b: ${\text{cams}}_{B_{b}}\in \left[2,5\right]$

${\mathrm{ALL}}_{\text{CAMS}}\leftarrow {\text{cams}}_{B}$

end for

$${\text{node}}_{\text{cams}\_\text{matrix}}\leftarrow \text{concat}\left({\mathrm{ALL}}_{\text{CAMS}}\right)$$

$S\leftarrow \text{mean}\left({\text{node}}_{\text{cams}\_\text{matrix}},\text{aixs}=0\right)$

return S

### 2.9 Comparison With Existing Methods

#### 2.9.1 Comparison With Graph Contrastive Learning (GCL) Methods

Most existing GCL methods rely on destructive graph augmentations such as node dropping and edge perturbation to generate contrastive views. However, these operations often destroy the inherent neurobiological plausibility of brain functional networks. Additionally, traditional GCL requires large volumes of additional unlabeled real data to achieve satisfactory performance, which remains a major bottleneck in MDD research due to high clinical heterogeneity and limited data sharing practices.

In contrast, Uni‐GCL completely abandons traditional destructive graph augmentations. Instead, it introduces a Universum‐guided contrastive learning paradigm that utilizes mixup‐generated synthetic samples as negative contrastive views. This strategy not only preserves the topological and functional integrity of brain networks but also eliminates the dependence on extra unlabeled real data, providing a tailored solution to the pervasive data scarcity challenge in clinical neuroimaging research.

#### 2.9.2 Comparison With Traditional Universum Learning

Traditional Universum learning approaches typically employ out‐of‐domain samples as Universum data, which are often irrelevant to the target task and may introduce unintended biases. Furthermore, very few prior studies have explored the application of Universum learning to graph neural networks for MDD brain network classification. The proposed Uni‐GCL generates in‐domain Universum samples via graph mixup, which strictly preserves the topological structure and functional characteristics of real human brain networks. These task‐relevant Universum samples provide more effective inter‐class constraints for contrastive learning without introducing external biases, significantly enhancing the model's generalization ability under limited labeled data conditions.

#### 2.9.3 Comparison With GCN Explainability Methods

While Grad‐CAM has been widely adopted in convolutional neural networks (CNNs) and static graph convolutional networks (GCNs), its application to spatio‐temporal graph convolutional networks (ST‐GCNs) for clinical neuroscience remains in its early stages. Most existing GCN explainability methods only provide node‐level importance scores for static graphs and lack both neurobiological constraints and rigorous causal validation mechanisms.

Our adapted graph Grad‐CAM addresses these limitations with three unique features: (1) It simultaneously quantifies the importance of both spatial brain regions and temporal dynamics, capturing the dynamic functional connectivity patterns that are characteristic of MDD; (2) It incorporates brain network topological constraints to ensure that the identified important regions align with established neurobiological knowledge; (3) It is validated through rigorous leave‐region‐out causal experiments, providing definitive evidence that the Grad‐CAM‐derived regions of interest (ROIs) are indeed the primary drivers of the model's diagnostic predictions.

## 3 Result

### 3.1 Classification Study

In REST‐MDD dataset, we pre‐trained the Uni‐GCL model at site 20 and then fine‐tuned and tested it at site 1 and site 21. In Table 2, we report the classification results of HC and MDD by Uni‐GCL, no confusion method, convolutional neural network (CNN), graph convolutional network (GCN), spatiotemporal graph convolutional network, respectively.

**TABLE 2:** Cross‐site model performance on REST‐MDD.

Method		ACC	AUC	REC	PRE	F1
Site 20‐>Site 21	CNN	0.58 (0.09)	0.62 (0.10)	0.62 (0.23)	0.65 (0.11)	0.60 (0.15)
	GCN	0.57 (0.06)	0.63 (0.09)	0.50 (0.14)	0.64 (0.03)	0.55 (0.10)
	ST‐GCN	0.60 (0.16)	0.64 (0.18)	0.61 (0.13)	0.65 (0.17)	0.63 (0.15)
	UCGL	0.63 (0.11)	0.65 (0.15)	0.63 (0.15)	0.68 (0.08)	0.65 (0.11)
	Mode	0.55 (0.001)	0.50 (0.00)	1.0 (0.00)	0.55 (0.001)	0.71 (0.001)
	Random	0.52 (0.04)	0.51 (0.04)	0.58 (0.06)	0.56 (0.04)	0.57 (0.05)
	Uni‐GCL	0.66 (0.05)	0.61 (0.16)	0.29 (0.25)	0.38 (0.31)	0.32 (0.27)
Site 20‐>Site 1	CNN	0.58 (0.09)	0.59 (0.12)	0.55 (0.15)	0.61 (0.11)	0.56 (0.09)
	GCN	0.58 (0.10)	0.66 (0.13)	0.54 (0.24)	0.61 (0.12)	0.54 (0.17)
	ST‐GCN	0.61 (0.09)	0.68 (0.11)	0.56 (0.15)	0.61 (0.10)	0.58 (0.13)
	UCGL	0.62 (0.14)	0.66 (0.16)	0.60 (0.17)	0.62 (0.13)	0.61 (0.15)
	Mode	0.50 (0.002)	0.50 (0.00)	0.20 (0.40)	0.10 (0.20)	0.13 (0.27)
	Random	0.49 (0.04)	0.49 (0.04)	0.48 (0.05)	0.49 (0.04)	0.49 (0.03)
	Uni‐GCL	0.67 (0.07)	0.67 (0.09)	0.65 (0.11)	0.69 (0.09)	0.66 (0.09)

*Note:* Classification results in terms of mean (standard deviation).

From Table 2, we can observe that the overall performance of Uni‐GCL is superior to other methods. The possible reason is that Uni‐GCL introduces universum samples through the mixup, improving the robustness and generalization ability of the model. It can extract richer and more transferable functional magnetic resonance imaging features without any annotated information, improving the classification performance of downstream tasks. For the Site20 → Site21 transfer, Uni‐GCL attains competitive overall accuracy but relatively low precision and recall for the MDD class. Here the model is pretrained on Site 20 and fine‐tuned with only a small labeled subset of Site 21. Due to residual inter‐site distribution differences and the limited target‐site supervision, the resulting classifier tends to adopt a conservative decision boundary on Site 21, correctly identifying many controls while missing some atypical MDD cases. The large standard deviations of the class‐wise metrics further indicate instability under this particularly challenging configuration. This observation highlights cross‐site calibration as an open problem and motivates future work on improved domain adaptation and cost‐sensitive training, rather than contradicting the overall effectiveness of Uni‐GCL.

To verify the effectiveness of our method, we applied it to the ADNI database for relevant validation. Detailed results can be found in Table A2 and Table A3 of the appendix.

### 3.2 Discriminative Brain Regions for MDD Identification

**FIGURE 2:**
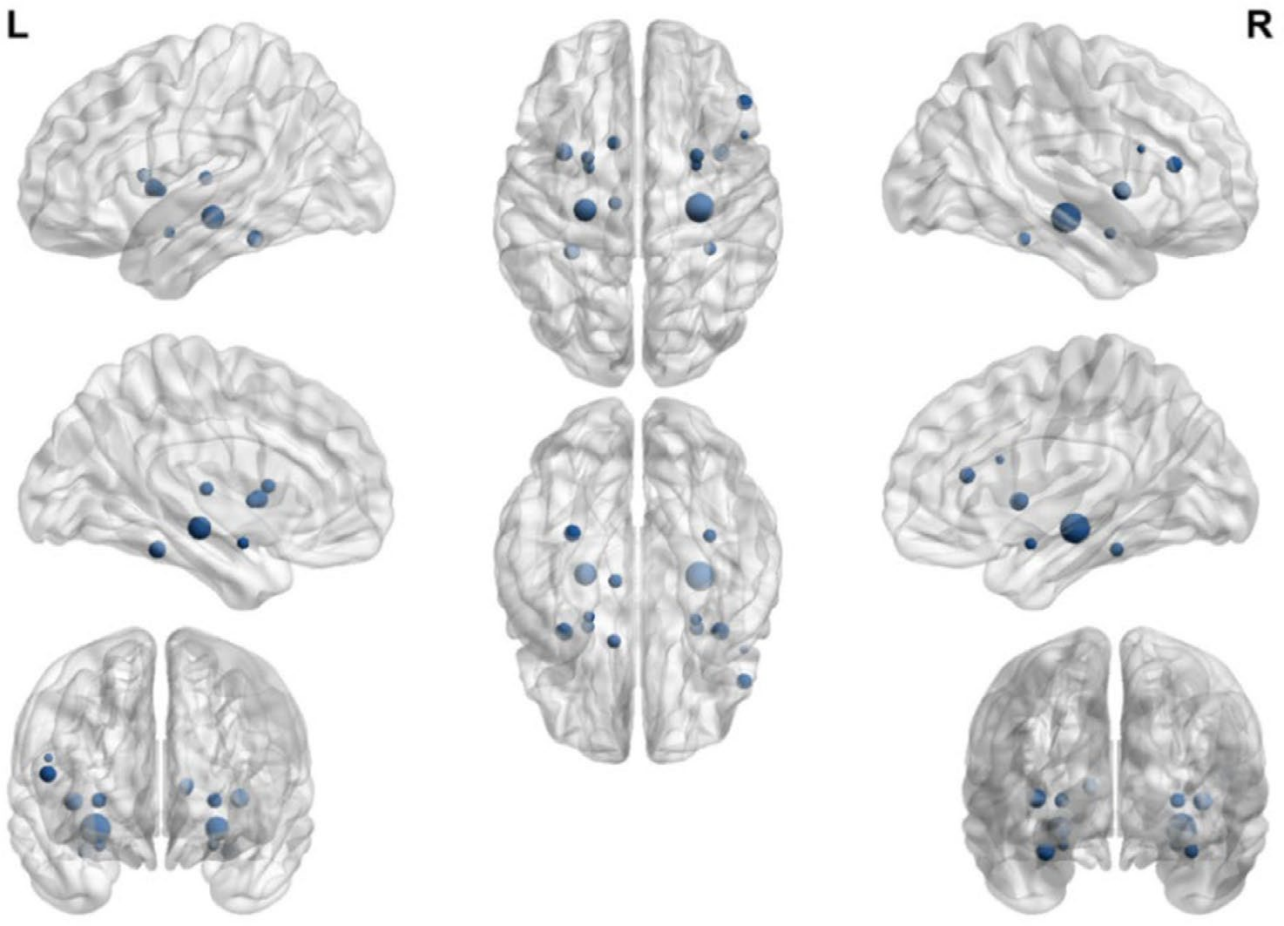
The most discriminative brain regions in identifying MDD (the size of the points represents the importance of each brain region, with larger points indicating greater significance).

To identify brain regions that contribute most to the MDD diagnosis, we compute node‐level Grad‐CAM scores on the model trained at Site 20 (Figure 2). For each subject, we back‐propagate the pre‐softmax class logit through the last graph/temporal block that still preserves node resolution, obtain a Grad‐CAM heatmap over nodes, and then average the heatmaps across subjects within the site to yield an ROI‐wise importance vector. For visualization, the node sizes are linearly mapped to the range 2–5 and we highlight the top 15% nodes (≈14/90 under AAL90). The same procedure applied to Site 21 and Site 1 reveals qualitatively similar spatial patterns. Full per‐node scores are provided in the Appendix.

In Table 3, the brain regions associated with MDD and their corresponding sub‐networks are presented for sites 20, 21, and 1. The training output of the model includes the activation values of the nodes, and then we search for the 14 brain regions with the highest contribution based on the activation values. This activation value represents the activation value after mapping, and the larger the value, the greater the contribution to MDD diagnosis.

**TABLE 3:** The top 14 contributing brain regions on Sites 1, 20, and 21.

Brain region	Sub‐network	Activation_value
HIP.R	—	5
HIP.L	—	4.068975
INS.L	Uncertain/Salience	3.374327
INS.R	Sensory/somatomotor mouth/Cingulo‐opercular task control/Uncertain/Salience	3.362881
FFG.L	Uncertain/Default mode/Visual	3.201759
IFGtriang.R	Ventral attention/Fronto‐parietal task control/Salience	3.069431
PUT.R	Subcortical	2.83433
CAU.L	—	2.782717
THA.L	Subcortical	2.768428
PUT.L	Subcortical	2.741344
FFG.R	Visual/Uncertain/Default mode	2.687069
AMYG.R	—	2.472371
AMYG.L	—	2.445723
IFGoperc.R	—	2

### 3.3 Leave‐Region‐Out Validation of Grad‐CAM Importance

To validate the robustness of the Grad‐CAM‐derived node‐wise importance, we conducted a targeted leave‐region‐out experiment on the Site 20 → Site 1 setting. We first selected the top 14 ROIs according to their Grad‐CAM sensitivity and then, for each ROI, removed its BOLD time series from all subjects and re‐trained the Uni‐GCL model with the same hyper‐parameters as in the main experiment. As summarized in Table 4, removing any of these ROIs generally leads to a decrease in ACC, AUC, and *F*1 compared to the full model using all ROIs, and removing all 14 ROIs simultaneously causes a more pronounced drop. This consistency between Grad‐CAM importance and leave‐region‐out degradation supports the relevance of the highlighted regions for the MDD‐HC classification.

**TABLE 4:** Leave‐region‐out analysis of Grad‐CAM‐identified ROIs on Site 20→Site 1.

	Brain region	ACC	AUC	REC	PRE	*F*1
Site 20‐>Site 1	Uni‐GCL	0.67 (0.07)	0.67 (0.09)	0.65 (0.11)	0.69 (0.09)	0.66 (0.09)
	HIP.R	0.62 (0.10)	0.60 (0.12)	0.72 (0.11)	0.60 (0.08)	0.65 (0.07)
	HIP.L	0.63 (0.11)	0.57 (0.11)	0.61 (0.11)	0.64 (0.11)	0.62 (0.10)
	INS.L	0.64 (0.06)	0.59 (0.08)	0.61 (0.10)	0.66 (0.09)	0.62 (0.06)
	INS.R	0.62 (0.10)	0.65 (0.09)	0.56 (0.13)	0.65 (0.12)	0.59 (0.11)
	FFG.L	0.62 (0.05)	0.63 (0.06)	0.55 (0.25)	0.65 (0.06)	0.55 (0.17)
	IFGtriang.R	0.63 (0.07)	0.63 (0.10)	0.63 (0.20)	0.63 (0.04)	0.62 (0.10)
	PUT.R	0.62 (0.06)	0.60 (0.05)	0.58 (0.12)	0.63 (0.06)	0.60 (0.06)
	CAU.L	0.63 (0.03)	0.59 (0.05)	0.65 (0.08)	0.62 (0.02)	0.63 (0.04)
	THA.L	0.63 (0.04)	0.61 (0.10)	0.58 (0.11)	0.66 (0.10)	0.61 (0.05)
	PUT.L	0.66 (0.04)	0.61 (0.07)	0.65 (0.17)	0.68 (0.09)	0.64 (0.07)
	FFG.R	0.64 (0.05)	0.62 (0.03)	0.69 (0.05)	0.63 (0.06)	0.66 (0.05)
	AMYG.R	0.61 (0.07)	0.61 (0.10)	0.58 (0.07)	0.63 (0.08)	0.60 (0.04)
	AMYG.L	0.66 (0.10)	0.65 (0.12)	0.63 (0.08)	0.68 (0.11)	0.65 (0.08)
	IFGoperc.R	0.64 (0.05)	0.62 (0.07)	0.68 (0.06)	0.64 (0.05)	0.65 (0.05)
	All (remove)	0.60 (0.06)	0.60 (0.09)	0.65 (0.08)	0.61 (0.07)	0.62 (0.05)

### 3.4 Relation to Other Graph Explainability Methods

Our Grad‐CAM‐like procedure provides a simple, class‐specific saliency score for each node by leveraging the gradients of the MDD logit with respect to the last GCN layer. Compared with optimization‐based approaches such as GNNExplainer, which learn instance‐wise masks over nodes and edges, and integrated‐gradients‐based attributions that require multiple evaluations along an interpolation path, our method is lightweight, deterministic, and easily scalable to the large number of subjects considered here. This makes it suitable for our purpose of validating whether Uni‐GCL predominantly relies on neurobiologically plausible regions. We note, however, that our analysis focuses on node‐level importance and does not explicitly account for edge‐level contributions. A more comprehensive comparison with dedicated graph explainability frameworks is an interesting direction for future work.

### 3.5 Brain Connection Analysis

**FIGURE 3:**
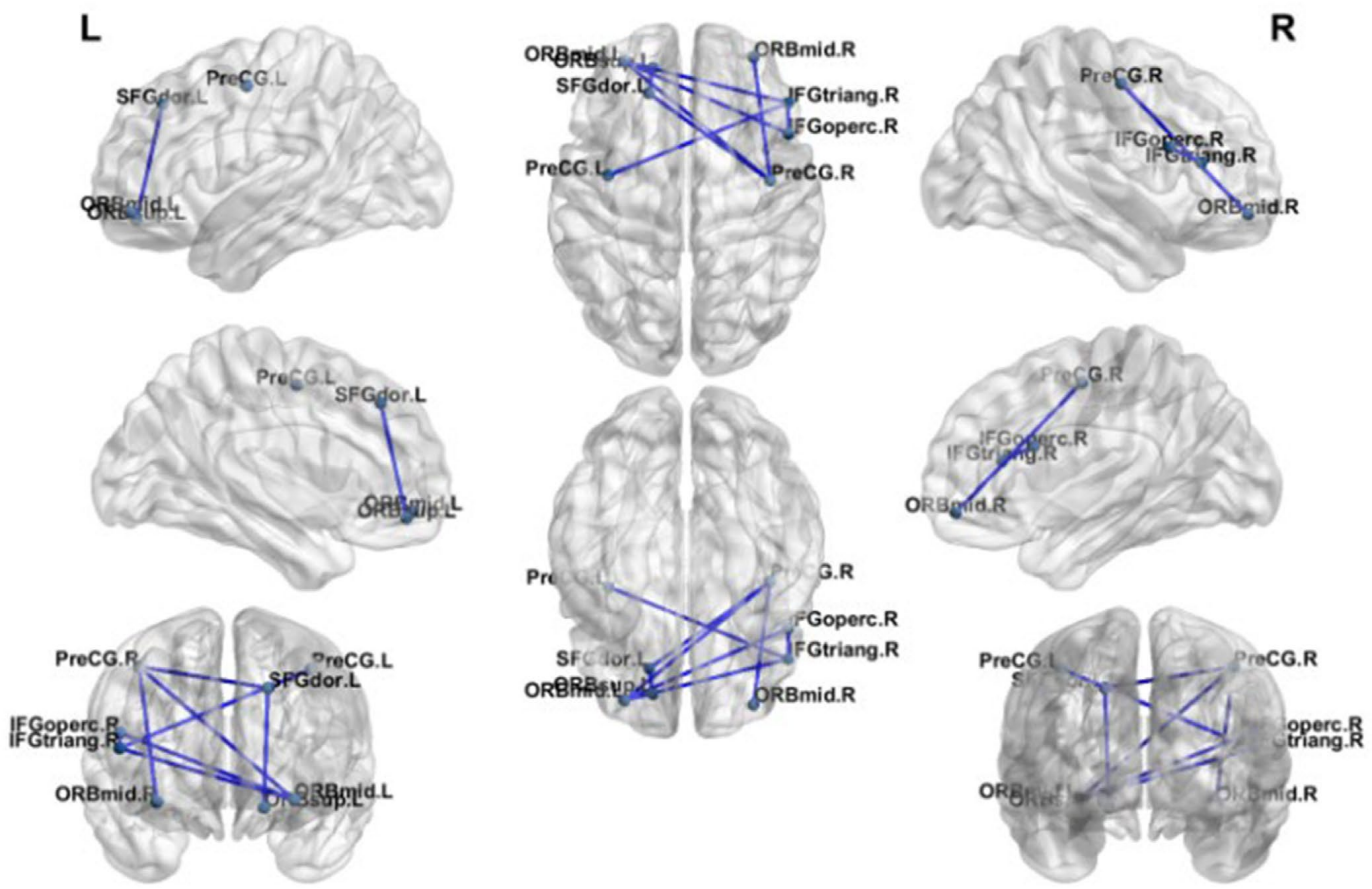
Significant brain connectivity differences for MDD versus HC.

**FIGURE 4:**
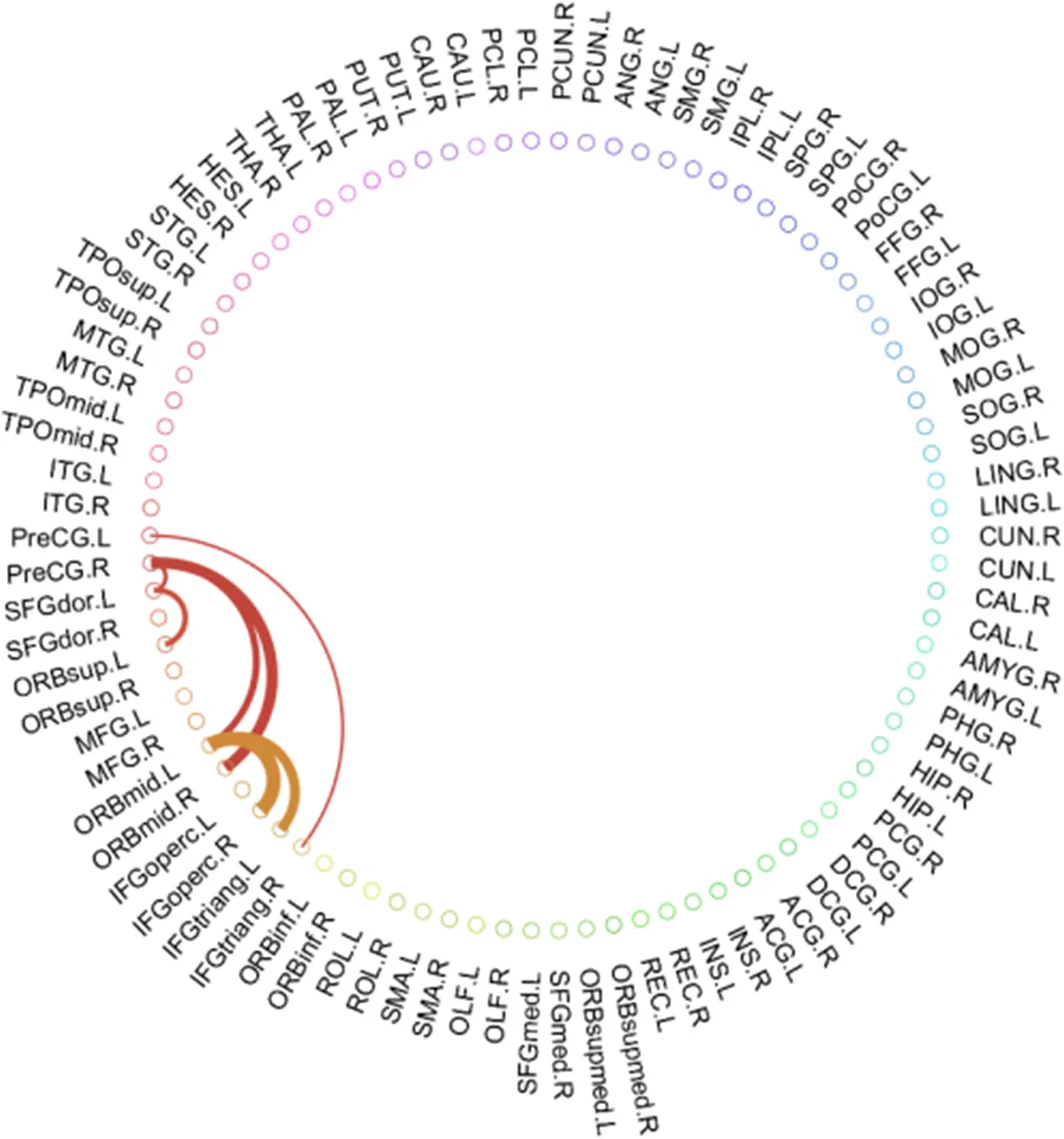
Significant brain connectivity differences annular chart.

To further analyze the functional connectivity changes in the most discriminative brain regions, we conducted a two‐sample *t*‐test between MDD patients and healthy controls to identify significant differences in brain connectivity, with a significance level set at *p* < 0.05. The mean connection value and corresponding *p*‐values from the *t*‐tests for brain regions showing significant differences between MDD patients and controls are summarized in Table 5. Figures 3 and 4 depict the significant differences in brain connectivity between MDD and control samples, where the thickness of the lines represents the strength of connections between brain regions. By comparing the connectivity strengths of brain regions with significantly differential connectivity patterns between MDD patients and healthy controls, we observed that most connectivity strengths in MDD patients were weaker than those in the control group. Importantly, these edge‐wise *t*‐tests were not corrected for multiple comparisons and are reported only as exploratory, descriptive summaries of connectivity differences. They are not used for formal statistical inference, and none of our main conclusions rely on these uncorrected statistics. The complete results for the 90 brain regions can be found in Table A5 of the appendix.

**TABLE 5:** Significant differences in brain connectivity on REST‐MDD.

ROI	ROI	Mean (MDD)	Mean (HC)	*p*
PreCG.L	IFGtriang.R	0.238	0.271	0.015
PreCG.R	SFGdor.L	0.251	0.289	0.006
PreCG.R	ORBmid.L	0.291	0.338	0.001
PreCG.R	ORBmid.R	0.357	0.393	0.015
SFGdor.L	ORBsup.L	0.257	0.285	0.044
ORBmid.L	IFGoperc.R	0.381	0.412	0.044
ORBmid.L	IFGtriang.L	0.334	0.384	0.001

### 3.6 Sensitivity Analysis

**FIGURE 5:**
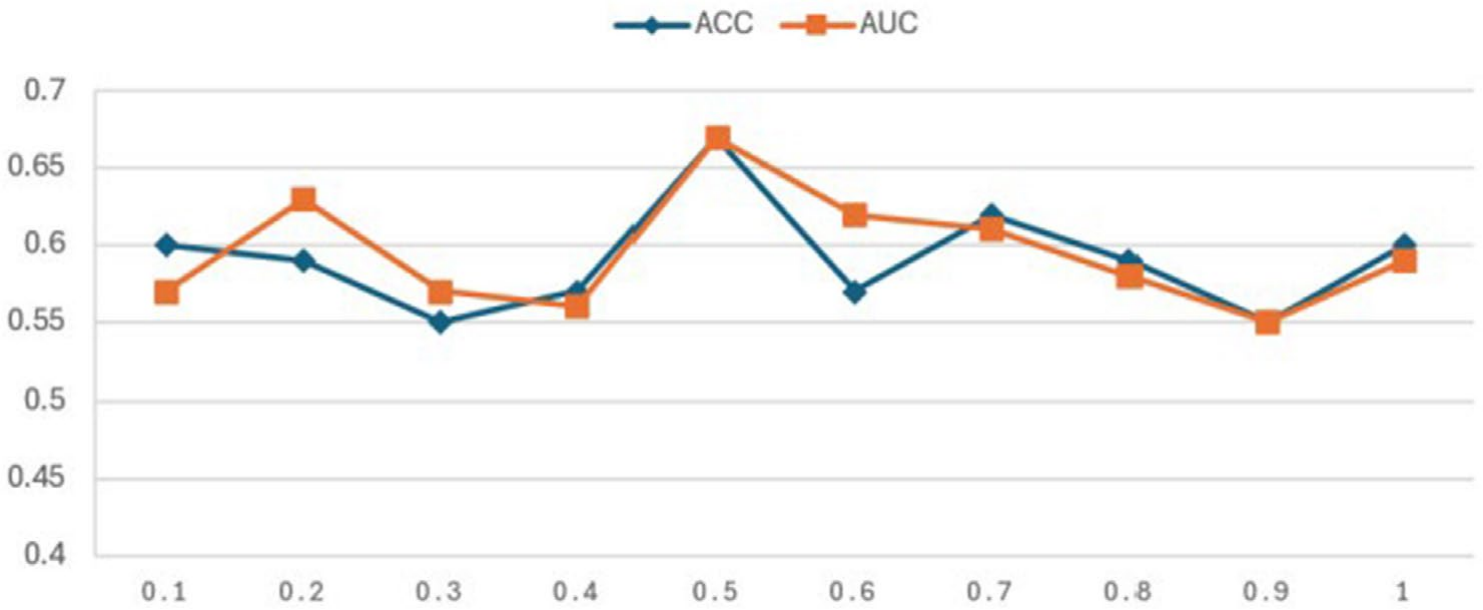
The impact of mixup parameter λ (i.e., Site 20‐>Site 1).

We further investigated the influence of the mixed coefficient λ the cross‐site setup (Site 20 → Site 1). Firstly, in the Appendix, we present a brief analysis based on information entropy, indicating that when λ=0.5, the label entropy of the mixed sample reaches its maximum, corresponding to the situation where the contributions of the two samples are equal. At this time, the generated sample has the highest uncertainty. It is more in line with the universum data setting based on mixup. Subsequently, in the cross‐site experiment, we conducted a grid search on $\lambda \in \left\{0.1,0.2,\ldots ,1.0\right\}$, and the results are shown in Figure 5. For detailed results, please refer to Table A4 in the appendix. Experiments show that the model performance varies significantly with λ: extremely unbalanced mixing coefficients can lead to a decline in metrics such as ACC and AUC, while λ=0.5 achieves the best overall performance in ACC, AUC, REC, PRE, and *F*1. Based on theoretical derivation and empirical results, we consider λ=0.5 as the recommended setting with a fundamental basis and stable effect in this framework. Detailed data results can be found in Table A4 of the appendix. In addition, we also considered how the similarity of positive and negative samples changes with the mixing parameter λ. The results can be found in Figure A3 of the appendix.

### 3.7 Ablation Experiment

To clarify the role of each component in Uni‐GCL, we conducted an ablation study under the same cross‐site assessment protocol. We consider the following three variants, all of which share the same backbone architecture and training/testing split:

Super gcl (supervised baseline only), the encoder has the same structure as Uni‐GCL, but is trained from scratch using a linear classifier with cross‐entropy loss. No two‐level fMRI augmentation, no contrast pre‐training, no cosmic mixing. This variant represents a setting where both the contrastive learning component and the time enhancement have been removed. UCGL, this model adopts self‐supervised contrastive pre‐training based on two‐level fMRI augmentation and then undergoes supervised fine‐tuning, without using hybrid‐generated full‐sum samples. In our framework, UCGL can be regarded as Uni‐GCL without the universum hybrid module, and thus can be considered as a natural dissolution of the proposed universum component. For Uni‐GCL (complete model), the method we proposed combines contrastive pre‐training based on two‐level fMRI augmentation with universum samples and subsequent supervised fine‐tuning.

**FIGURE 6:**
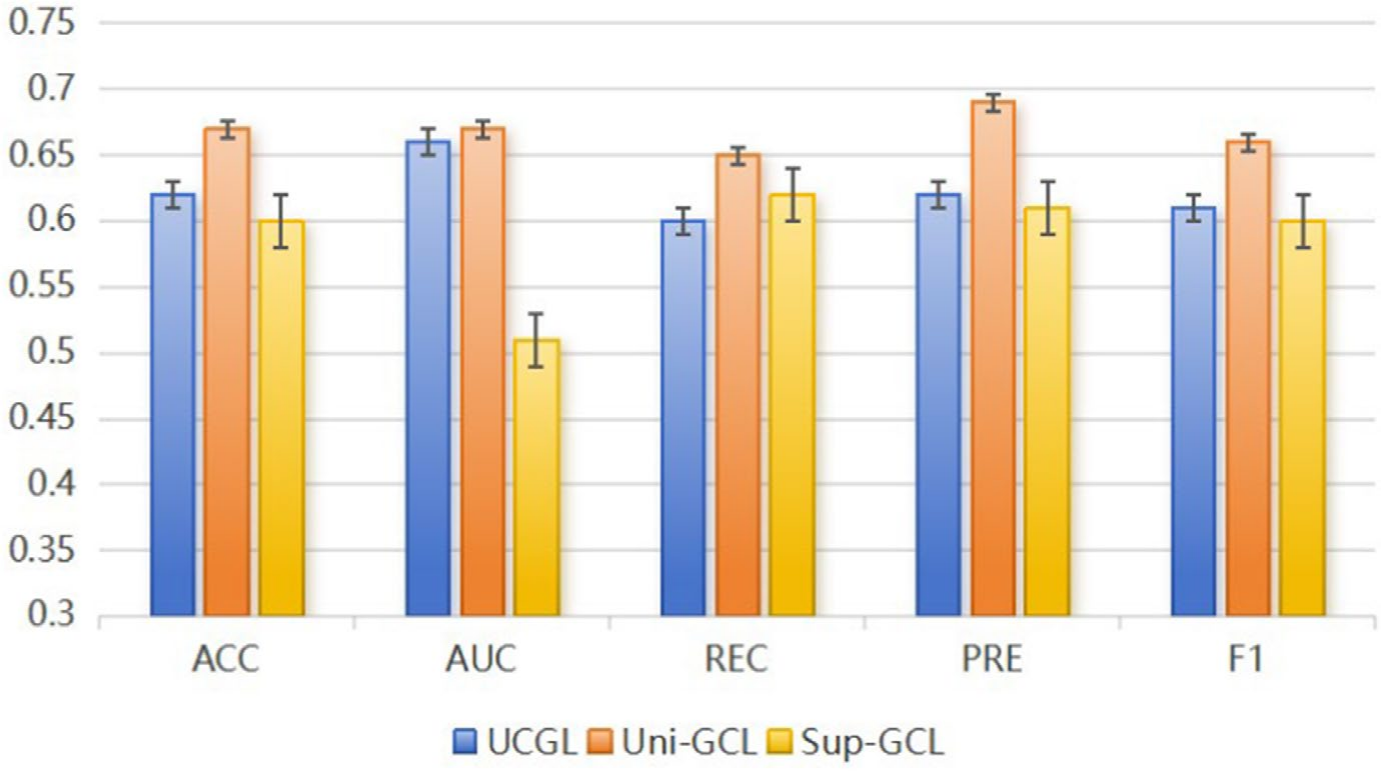
Ablation study of Uni‐GCL: Effect of two‐level fMRI augmentation, contrastive pretraining and universum mixup on cross‐site MDD classification (Site 20‐>Site 1).

**FIGURE A2:**
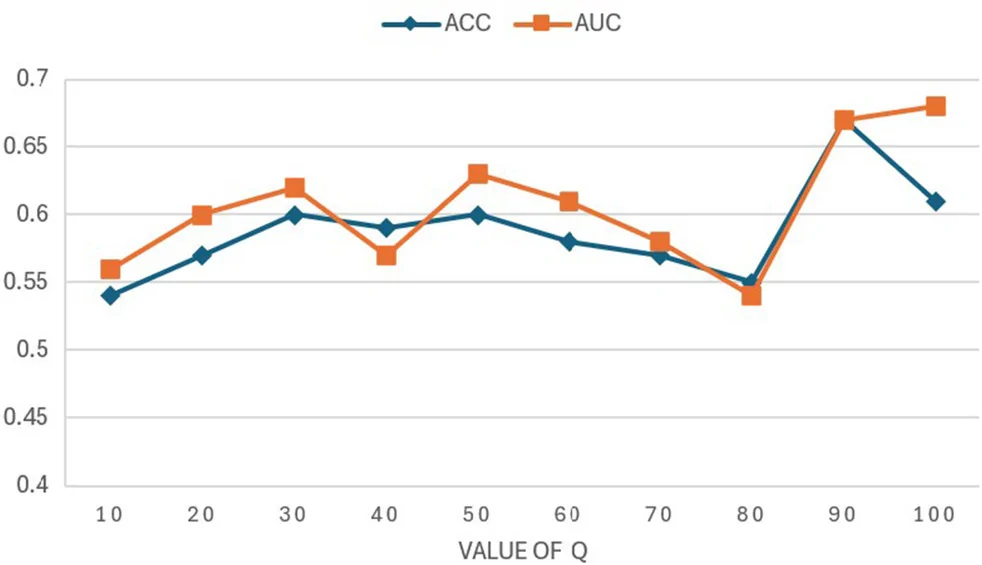
Performance of different window slice lengths on REST‐MDD (Site 20‐>Site 1).

**FIGURE A3:**
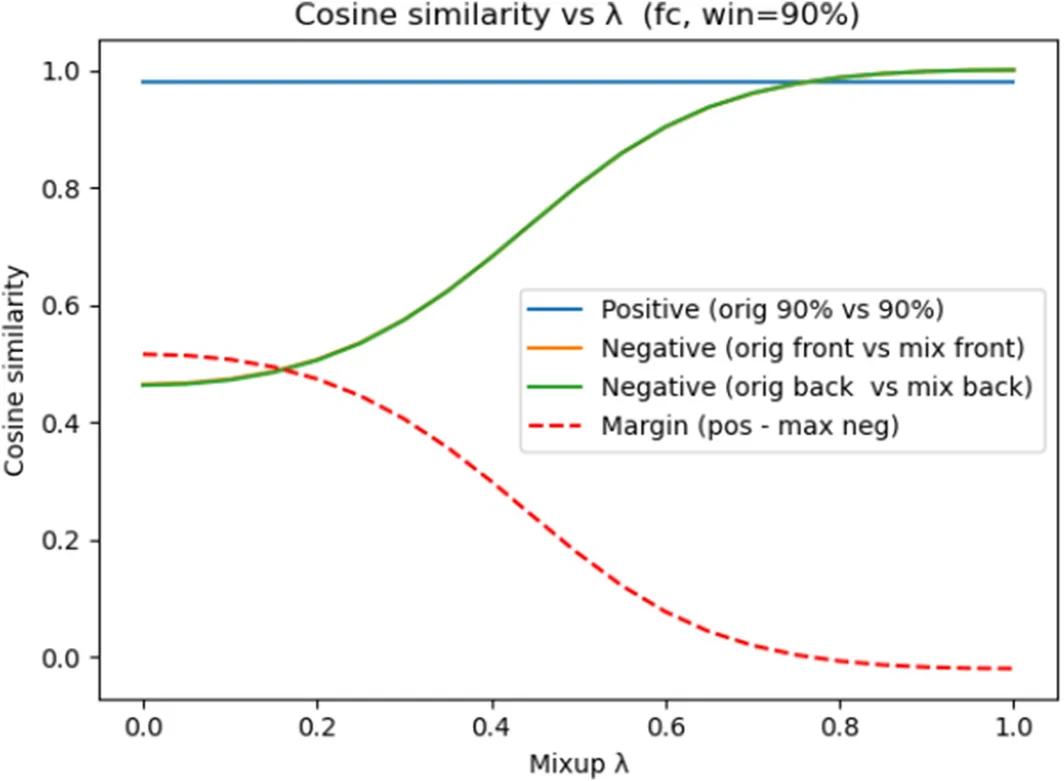
Cosine similarity versus λ.

The results are summarized in Figure 6 and Table 6. Compared with su‐gcl, UCGL continuously improves ACC, AUC, and *F*1, which indicates that two‐level fMRI augmentation and contrastive pre‐training provide more transferable representations than purely supervised training of encoders. Furthermore, Uni‐GCL achieved additional benefits over UCGL in all indicators, indicating that introducing the universum blend on top of the comparison framework brings complementary advantages. In conclusion, these fading provide a step‐by‐step validation for our design: from the supervised baseline (su‐gcl), to contrastive learning with two‐level fMRI augmentation (UCGL), and finally to the complete Uni‐GCL mixed with universum. In addition, we also considered the impact of the sliding window length on the model. The results can be found in Figure A2 of the appendix.

**TABLE 6:** Ablation study of Uni‐GCL (Site 20‐>Site 1).

Method	ACC	AUC	REC	PRE	F1
UCGL	0.62 (0.14)	0.66 (0.16)	0.60 (0.17)	0.62 (0.13)	0.61 (0.15)
Sup‐GCL	0.60 (0.07)	0.51 (0.08)	0.62 (0.11)	0.61 (0.10)	0.60 (0.06)
Uni‐GCL	0.67 (0.07)	0.67 (0.09)	0.65 (0.11)	0.69 (0.09)	0.66 (0.09)

## 4 Discussion

### 4.1 Main Findings

In this study, we proposed Uni‐GCL, a Universum‐inspired graph contrastive learning framework for identifying major depressive disorder from resting‐state functional brain networks. Across multiple imaging sites, Uni‐GCL consistently outperformed existing state‐of‐the‐art approaches, particularly in cross‐site generalization tasks, underscoring the effectiveness of incorporating boundary‐aware information into self‐supervised representation learning.

To identify brain regions that contribute most to the MDD prediction of Uni‐GCL [33], we computed node‐wise importance using a Grad‐CAM‐like strategy for each site and summarized the top 14 regions in Table 3 (FGoperc.R, CAU.L, FFG.R, AMYG.L, PUT.L, AMYG.R, PUT.R, THA.L, IFGtriang.R, FFG.L, INS.L, INS.R, HIP.L, HIP.R). Importantly, these regions do not emerge as isolated brain region but form coherent sub‐networks of large‐scale brain networks that have been consistently implicated in the pathophysiology of MDD. This distributed pattern suggests that Uni‐GCL relies on coordinated network‐level alterations rather than localized abnormalities, which is in line with contemporary systems‐level models of depression.

Specifically, hippocampal and amygdala regions (HIP.L/R, AMYG.L/R) constitute central components of the limbic system and have shown robust structural and functional alterations across multiple MDD cohorts. Abnormalities within these regions are closely associated with affective dysregulation, stress sensitivity, and memory‐related vulnerability, all of which are core features of depressive symptomatology. The consistent attribution of bilateral hippocampal and amygdalar nodes by Uni‐GCL indicates that the learned representations capture limbic network signatures linked to emotional memory processing, rather than relying on isolated regional effects [34, 35, 36].

Moreover, striatal and thalamic nodes (CAU.L, PUT.L/R, THA.L) form fronto‐striatal‐thalamic circuits involved in reward processing and action selection, where abnormal morphology and connectivity have been reported in depressed patients [37, 38]. Insular and inferior frontal regions (INS.L/R, IFGtriang.R, FGoperc.R) are core components of the salience and cognitive‐control networks; prior work has demonstrated insular dysfunction and altered salience–default mode coupling in MDD [39, 40, 41]. Fusiform regions (FFG.L/R), engaged in visual and socio‐emotional processing, have been associated with biased processing of affective stimuli in depression.

Taken together, these observations indicate that Uni‐GCL tends to assign higher importance to nodes within limbic, subcortical, salience, and visual networks that closely interact with the default mode network [42, 43], which is consistent with large‐scale network models of MDD highlighting disrupted interactions between default mode, salience, and fronto‐limbic circuits [44, 45, 46]. We emphasize that this analysis is descriptive and exploratory: it does not establish these 14 regions as uniquely pivotal for MDD, nor does it constitute a formal test of the superiority of our feature extraction mechanism or of subtle topological differences.

### 4.2 Limitations

Several limitations should be acknowledged. First, this study has a small sample size. Although we utilize sliding window strategy and mixup strategy for data augmentation, it is still difficult to fully overcome the problem of small sample data size. Second, the current analysis is based on static functional connectivity, and static representations may therefore obscure transient but clinically relevant interactions. Future work will extend Uni‐GCL to dynamic graph architectures to capture time‐resolved network reconfigurations and further elucidate the spatiotemporal organization of depressive brain states.

Another important limitation of this study is the insufficient in‐depth analysis of clinical confounding factors, including medication status, illness duration, and symptom severity. Although these factors may potentially alter the functional connectivity patterns of MDD patients and thus influence classification performance, the primary objective of the current work was to develop a novel Universum‐inspired graph contrastive learning framework to address the pervasive labeled data scarcity problem in neuroimaging research. Future studies will address this limitation in two ways. First, we will conduct targeted subgroup analyses on datasets with more comprehensive clinical information to disentangle the specific effects of medication, illness duration, and symptom severity on model performance. Second, we will develop confounder‐aware graph neural network architectures that can explicitly model and mitigate the impact of these confounding variables, thereby improving the generalizability and clinical utility of neuroimaging‐based MDD classification models.

## 5 Conclusion

This utilizes a mixup strategy to generate universum samples to alleviate the problem of dependence on annotated data. Combined with optimized Grad‐CAM technology, it accurately identifies 14 brain regions and their functional connectivity patterns that play a key role in the diagnosis of depression, such as FGoperc.R and HIP.L/R. This provides a new theoretical perspective on cortical sub‐cortical network interaction abnormalities for the study of depression pathological mechanisms. At the same time, its self‐supervised graph contrastive learning paradigm provides new ideas for the diagnosis and analysis of mental illnesses.

## Funding

This work was supported by the National Natural Science Foundation of China (Grants 62306051, 82227807, 82102023, 81830059, 6230073196, and 62481540175), Research Project of Shanghai Municipal Health Commission (Grants 20224Y0340 and 2022JC017), Scientific and the Technological Research Program of Chongqing Municipal Education Commission (Grant KJQN202300718), Taishan Scholars Foundation of Shandong Province (Grant tsqn202507225), the Clinical Research Plan of SHDC (Grant SHDC2020CR1038B), the Shanghai Municipal Health and Family Planning Commission Smart Medical Special Research Project (Grant 2018ZHYL0105), Joint Training Base Construction Project for Graduate Students in Chongqing (Grant JDLHPYJD2021016), the Science and Technology Commission of Shanghai Municipality (Grant 25SF1908900), and the Clinical Research Project of Tongji Hospital of Tongji University (Grants ITJ(ZD)2301 and ITJ(QN)2312).

## Ethics Statement

This study exclusively used two publicly available neuroimaging datasets: ADNI and REST‐meta‐MDD. All necessary ethical approvals and informed consent procedures were completed by the original data providers at the time of data collection. No additional ethical approval was required for the secondary data analysis performed in this research.

## Conflicts of Interest

The authors declare no conflicts of interest.

## Data Availability

The data that support the findings of this study are available on request from the corresponding author. The data are not publicly available due to privacy or ethical restrictions.

## Materials

In ADNI [47], there are 563 scans from 174 subjects, including 154 HC, 165 early mild cognitive impairment (EMCI), 145 late MCI (LMCI), and 99 AD scans. We do not consider site information in ADNI since this database uses harmonized scanning protocols. And the demographic and clinical information is given in Table A1.

**TABLE A1:** Demographic and clinical information of subjects from ADNI datasets.

Dataset	Category	Scan	Gender (M/F)	Age (mean ± SD)
ADNI	AD	99	55/44	75.04 ± 7.71
	LMCI	145	95/50	71.99 ± 7.67
	EMCI	165	73/92	72.03 ± 7.26
	HC	154	67/87	75.36 ± 6.16

For each fMRI, TR is 2200–3100 ms, TE = 30 ms, time = 7 min, the number of time points = 140, the in‐plane image resolution is 2.29–3.31 mm, slice thickness = 3.31 mm. All rs‐fMRI scans in ADNI are pre‐processed with FSL FEAT [48]. We first discard the first 3 volumes for magnetization stabilization and then process the remaining 137 volumes with the standard pipeline, including slice timing correction, head motion estimation, bandpass filtering (0.015–0.15 Hz), and regression of nuisance covariates (i.e., head motion parameters, white matter, and cerebrospinal fluid). Then, we perform skull dissection based on T1‐weighted MRI and align the fMRIs of skull dissection into MNI space. The resulting data are further spatially smoothed by Gaussian kernel with full‐width‐at‐half‐maximum (FWHM) of 6 mm. Finally, we extract the average rs‐fMRI time series of 116 predefined ROIs based on the AAL atlas [29].

## Methods

### Two‐Level fMRI Augmentation and Feature Extraction

Specifically, we perform data augmentation on all samples in the training dataset and the universum samples generated through the mixup [21] for each sample. $X={\left(x_{1},\ldots ,x_{N}\right)}^{T}\in R^{N\times T}$ as the rs‐fMRI time series of a subject, where $x_{n}=\left(x_{1,n},\ldots ,x_{T,n}\right)\in R^{T}$ denotes the mean BOLD signal intensity for the n‐th region interest (ROI), $x_{t,n}$ denotes the mean BOLD signal intensity of the nth ROI at time node *t*, *T* corresponds to the count of time points in the unaltered BOLD signal, while *N* = 116 represents the total number of ROI. By selecting a slice length *S*, the initial and final *S* time points of the time series can be truncated to generate two new BOLD signal samples. Mathematically, these enhanced BOLD signals can be represented as $X_{i}=\tau_{i}\left(X\right)\in R^{N\times S}\left(i=1,2\right)$, where $\tau_{1}\left(X\right)$ and $\tau_{2}\left(X\right)$ denote the front and back *S* column of the original sample generated via a window‐slicing strategy.

### Graph Adjacency Matrix

For each view, we construct a fixed ROI‐wise functional connectivity graph used by the ST‐GCN [32]. Let $x_{i}\left(t\right)$ denote the BOLD signal of the ith ROI across all time points and all training subjects (after temporal windowing or mixup, depending on the view). We compute a group‐level connectivity matrix:

(A1)
$$A_{\mathrm{ij}}=|\text{corr}\left(x_{i},x_{j}\right)|,A_{\mathrm{ii}}=1,$$

without applying any hard threshold, yielding a dense weighted graph. The final adjacency used in the ST‐GCN is the symmetrically normalized matrix.

(A2)
$$\tilde{A}=D^{-\frac{1}{2}}AD^{-\frac{1}{2}},D_{\mathrm{ii}}=\sum_{j}A_{\mathrm{ij}},$$

This matrix is pre‐computed from the training data (e.g., Site 20 in the cross‐site setting) and kept fixed for all subjects and target sites.

### Parallel fMRI Feature Extraction

Considering the fact that the topological structure of functional brain networks is induced by spatial and temporal correlation, we use ST‐GCN as the feature extractor to learn spatiotemporal features of fMRI data (see Figure A1). In this way, rich fMRI representations can be learned without the need to input any class labels for fMRI. In this work, two enhanced views $X_{i}\left(i=1,2\right)$ are input into parallel ST‐GCN feature extractors, respectively. Taking a single view as an example, we first construct spatiotemporal maps for each subject based on enhanced fMRI $X_{i}$.

To simulate the functional connectivity network of the brain, a spatiotemporal map was constructed for each view. $G_{i}\left(V_{i},E_{i}\right)$ as a spatiotemporal graph was constructed for an enhanced view $X_{i}\left(i=1,2\right)$. A set of nodes/ROIs defined at S time points as follows:

(A3)
$$V_{i}=\left\{\left(v_{t_{i,n}}\right)|t_{1}=1,\ldots ,S;t_{2}=T-S+1,\ldots ,T;n=1,\ldots ,N\right\},$$

$E_{i}$ is defined as a set of edges that signify the interconnections among auxiliary nodes across both temporal and spatial domains. In more detailed terms, for each ROI and moment in time, an edge in the temporal graph links the pertinent node to the same ROI at successive time points. Concurrently, at a given time point and ROI, the spatial graph's edges join all nodes present at that same moment, with the weights of these edges being determined by the Pearson correlation coefficients between the different ROIs.

In the ST‐GCN model, for the purpose of deriving brain connectivity patterns and temporal information from fMRI time series data, this study employs spatiotemporal graph convolution (STGC) on every input spatiotemporal graph. To define convolution on $G_{i}$, we use $f_{\text{in}}\left(v_{t_{i,n}}\right)$ to represent the node features $v_{t_{i,n}}$ and $B\left(v_{t_{i,n}}\right)$ to represent the spatiotemporal domain of points $v_{t_{i,n}}$ as follows:

(A4)
$$B\left(v_{t_{i,n}}\right)=\left\{v_{q,m}|d\left(v_{t_{i,n}},v_{t_{i,m}}\right)\leq K,|q-t_{i}|\leq \left\lfloor L/2\right\rfloor \right\},$$

Here, *K* denotes the dimension of the spatial kernel, *L* signifies the extent of the temporal kernel, and $d\left(\cdot ,\cdot \right)$ denotes the distance between two nodes. The application of the STGC operation on a node $v_{t_{i,n}}$ can be articulated through the following equation:

(A5)
$$f_{\text{out}}\left(v_{t_{i,n}}\right)\frac{1}{Z_{t_{i,n}}}\sum_{v_{q,m\in B\left(v_{t_{i,n}}\right)}}f_{\text{in}}\left(v_{q,m}\right)w\left(v_{q,m}\right),$$

Among them $Z_{t_{i,n}}$ is the normalization factor, $w\left(\cdot \right)$ is the spatial–temporal convolution kernel. Here, the spatial–temporal convolution kernel $w\left(\cdot \right)$ is approximated by decomposing it into spatial convolution kernel $W_{S}\in R^{C\times M}$ and temporal convolution kernel $W_{T}\in R^{M\times L}$. Specifically, assuming that $f_{t_{i}}\in R^{N\times C}$ represents the input features of N ROIs at time point $t_{i}$ in channel *C* and $A\in R^{N\times N}$ is a correlation matrix, the spatial convolution at time point $t_{i}$ is defined as:

(A6)
$$f_{t_{i}}^{\prime }=\wedge^{-\frac{1}{2}}\left(A+I\right)\wedge^{-\frac{1}{2}}f_{t_{i}}W_{s},$$

where $\wedge^{n,n}=\sum_{j}A^{n,j}+I$ is a diagonal matrix; $f_{t_{i}}^{\prime }\in R^{N\times M}$ indicates the output characteristics of the *M* channel. This article further performs temporal convolution on the obtained spatial features. Taking nodes $v_{n}$ as an example, the output after temporal convolution can be represented as:

(A7)
$$f_{n}^{\prime }⨂W_{T}\in R^{M\times S},$$

Among them $f_{n}^{\prime }\in R^{M\times S}$ are the features defined by nodes $v_{n}$ on temporal graphs, ⨂ denotes a standard 1D convolution.

In this study, three layers of STGC units are incorporated into each ST‐GCN. The initial layer receives the *C*‐channel feature as input, and each subsequent layer produces the M‐channel feature as output. The output from the final STGC layer is then directed to a global average pooling layer, which yields a 64‐dimensional graph‐level representation vector. Concisely, this representation vector can be algebraically represented as follows:

(A8)
$$z_{i}=F\left(X_{i}\right)\in R^{64},$$

Among them $F\left(\cdot \right)$ denotes the transformation function of the ST‐GCN feature extraction component.

MLP [49] predictor. For each view (e.g., $X_{1}$), this article also defines an MLP predictor $H\left(\cdot \right)$ for feature abstraction, which is applied to convert the output from one perspective or branch and align it with the other branch. Each MLP module comprises two fully‐connected layers, which contain 32 and 64 neurons, respectively. Taking $z_{i}\left(i=1,2\right)$ as input, the MLP predictor will generate a 64‐dimensional feature vector:

(A9)
$$p_{i}=H\left(z_{i}\right)=H\left(F\left(X_{i}\right)\right),$$

### Contrastive Learning

(A10)
$$\psi \left(z_{i},p_{j}\right)=-\frac{z_{i}}{∥z_{i}{∥}_{2}}\cdot \frac{p_{j}}{∥p_{j}{∥}_{2}},$$

where $∥\cdot {∥}_{2}$ represents a binomial. For these perspectives, we establish the following symmetric loss function to enhance the similarity between two entities of the same object and weaken the similarity between entities of different objects:

(A11)
$$\xi =\frac{1}{2}\left(\psi \left(z_{1},p_{2}\right)+\psi \left(z_{2},p_{1}\right)\right)-\frac{1}{4}\left(\psi \left(z_{1},{\hat{p}}_{1}\right)+\psi \left({\hat{z}}_{1},p_{1}\right)+\psi \left(z_{2},{\hat{p}}_{2}\right)+\psi \left({\hat{z}}_{2},p_{2}\right)\right),$$

From Equation (A9), $z_{i}$ and ${\hat{z}}_{i}\left(i=1,2\right)$ denote the feature representations of the original samples and Universum samples after data augmentation across two views, respectively. $p_{i}$ and ${\hat{p}}_{i}\left(i=1,2\right)$ denote the output of $z_{i}$ and ${\hat{z}}_{i}$ processed by a MLP.

Gradient stopping technique [50]. In line with the approach described by Chen and He, this study also employs a gradient stopping operation $g\left(\cdot \right)$ on each view within the pre‐trained model, which is a crucial step to avoid model degradation. Consequently, Equation (A8) can be restated as follows:

(A12)
$$\psi \left(g\left(z_{1}\right),p_{2}\right),$$

## Result

We pre‐trained the model using Site 20 data and then fine‐tuned it on the ADNI. Given that the ADNI dataset adopts a unified scanning protocol, we ignored site information in the experiment and focused on testing the classification tasks of Alzheimer's disease (AD) and normal control group (HC), mild cognitive impairment (LMCI), and early mild cognitive impairment (EMCI). The results are shown in Tables A2 and A3.

### Sensitivity Analysis

Here, a concise analysis is provided to illustrate the importance of setting the value of λ to 0.5. In the context of mix‐up, when blending data points with a mixing coefficient of λ, the resulting label should be $y=\left\{0,\ldots ,\lambda ,\ldots ,1-\lambda ,\ldots ,0\right\}$, where the placement of λ and 1−λ corresponds to the respective mixed data. Consequently, the information entropy of y can be computed as follows:

(A13)
$$H\left(y\right)=-\lambda \log\left(\lambda \right)-\left(1-\lambda \right)\log\left(1-\lambda \right),$$

To obtain the maximum value of $H\left(y\right)$, we derived the gradient relative to λ of $H\left(y\right)$:

(A14)
$$\frac{\partial H\left(y\right)}{\partial \lambda }=\log\left(\frac{1-\lambda }{\lambda }\right),$$

At that time λ=0.5, $\partial H\left(y\right)/\partial \lambda$ = 0. At this juncture, the maximum value of $H\left(y\right)$ is achieved, and the label vector y exhibits the greatest degree of uncertainty. Therefore, at that time λ=0.5, data based on mix‐up could be best described as the universal class of all data, since such samples are the most uncertain. Table A4 shows the impact of the value of λ on the results.

**TABLE A2:** Cross‐dataset model performance on ADNI for AD and HC.

Method	ACC	AUC	REC	PRE	F1
CNN	0.76 (0.13)	0.86 (0.14)	0.68 (0.05)	0.74 (0.18)	0.70 (0.11)
GCN	0.78 (0.13)	0.83 (0.15)	0.72 (0.11)	0.74 (0.18)	0.73 (0.14)
ST‐GCN	0.81 (0.13)	0.84 (0.16)	0.74 (0.23)	0.75 (0.16)	0.74 (0.19)
UCGL	0.84 (0.12)	0.90 (0.12)	0.79 (0.17)	0.80 (0.16)	0.79 (0.17)
Uni‐GCL	0.85 (0.11)	0.94 (0.07)	0.86 (0.13)	0.85 (0.15)	0.83 (0.17)

**TABLE A3:** Cross‐dataset model performance on ADNI for LMCI and EMCI.

Method	ACC	AUC	REC	PRE	F1
CNN	0.72 (0.09)	0.79 (0.13)	0.68 (0.09)	0.71 (0.10)	0.70 (0.09)
GCN	0.73 (0.13)	0.81 (0.17)	0.70 (0.13)	0.72 (0.15)	0.71 (0.14)
ST‐GCN	0.74 (0.07)	0.80 (0.11)	0.77 (0.07)	0.71 (0.07)	0.74 (0.07)
UCGL	0.77 (0.16)	0.81 (0.18)	0.77 (0.20)	0.75 (0.17)	0.76 (0.18)
Uni‐GCL	0.83 (0.11)	0.91 (0.07)	0.89 (0.14)	0.80 (0.13)	0.84 (0.23)

**TABLE A4:** The impact of mix‐up parameters λ for MDD (i.e., Site 20‐>Site 1).

λ	ACC	AUC	REC	PRE	F1
0.1	0.60 (0.08)	0.57 (0.11)	0.62 (0.13)	0.59 (0.08)	0.60 (0.09)
0.2	0.59 (0.06)	0.63 (0.06)	0.65 (0.13)	0.57 (0.05)	0.61 (0.08)
0.3	0.55 (0.03)	0.57 (0.07)	0.49 (0.22)	0.55 (0.04)	0.48 (0.18)
0.4	0.57 (0.06)	0.56 (0.13)	0.41 (0.20)	0.59 (0.08)	0.46 (0.17)
0.5	0.67 (0.07)	0.67 (0.09)	0.65 (0.11)	0.69 (0.09)	0.66 (0.09)
0.6	0.57 (0.04)	0.62 (0.07)	0.54 (0.13)	0.57 (0.04)	0.54 (0.09)
0.7	0.62 (0.09)	0.61 (0.05)	0.62 (0.17)	0.60 (0.09)	0.61 (0.13)
0.8	0.59 (0.09)	0.58 (0.08)	0.55 (0.09)	0.62 (0.14)	0.58 (0.08)
0.9	0.55 (0.08)	0.55 (0.10)	0.53 (0.11)	0.56 (0.08)	0.54 (0.07)
1.0	0.60 (0.05)	0.59 (0.05)	0.65 (0.08)	0.60 (0.06)	0.62 (0.04)

### Influence of Slice Length

In the pretext experiment, we used a sliding window slicing strategy to enhance fMRI data, with a slice length of *S* = *q*% *T* (i.e., slicing *q*% of the original BOLD signal), where *T* is the time point of the original BOLD signal [51]. In the pretext experiment, the value of *q* = 90. In order to investigate the effect of slice length, this study conducted comparative experiments on *q* values of 10, 20, 30, 40, 50, 60, 70, 80, 90 and 100, and reported the results of our Uni‐GCL in MDD and HC classification in Figure A2. The experiment was conducted on REST‐MDD, with the model pre‐trained on Site 20 and fine‐tuned on Site 1. In the case of *q* = 100 (i.e., retaining all BOLD signals), no data augmentation is required. From Figure A2, it can be seen when *q* < 50, UCGL cannot produce competitive performance, which may be due to the inability to effectively capture the spatiotemporal dependence of brain activity using too short BOLD signals. Uni‐GCL achieved optimal ACC and AUC results at *q* = 90.

### Significant Node for MDD Identification

Based on the Grad‐CAM theory [25], detailed information of each brain region is shown in Table A5, where value represents the result of mapping the activation value to a range of 2–5 after softmax operation. And this result is based on the AAL90 template.

### The Cosine Similarity of Positive and Negative Sample Pairs Varies With λ

We scan λ from 0 to 1 and calculate the cosine similarity of all positive and negative pairs to characterize how the separation between positive and negative similarities varies as a function of the mixing intensity. The experimental results are shown in Figure A3.

**TABLE A5:** The contribution of brain regions for MDD.

Site 21 ROI value		Site 20 ROI value		Site 1 ROI value	
HIP.R	5.000000	HIP.R	5.000000	HIP.R	5.000000
HIP.L	4.651335	HIP.L	4.561762	HIP.L	4.563335
INS.L	4.237847	INS.L	4.234788	INS.L	4.122675
INS.R	4.225951	INS.R	4.229401	INS.R	4.104360
FFG.L	4.176636	FFG.L	4.153560	FFG.L	3.958361
IFGtriang.R	4.110420	IFGtriang.R	4.091272	IFGtriang.R	3.927279
PUT.R	4.052574	PUT.R	3.980609	PUT.R	3.875253
CAU.L	4.015700	CAU.L	3.956315	CAU.L	3.871912
THA.L	3.944635	THA.L	3.949589	THA.L	3.838973
PUT.L	3.932777	PUT.L	3.936840	PUT.L	3.812424
FFG.R	3.913763	FFG.R	3.911293	FFG.R	3.779606
AMYG.R	3.883140	AMYG.R	3.810233	AMYG.R	3.778477
AMYG.L	3.838482	AMYG.L	3.797690	AMYG.L	3.542576
IFGoperc.R	3.564745	IFGoperc.R	3.587886	IFGoperc.R	3.478498
DCG.R	3.483296	DCG.R	3.490429	DCG.R	3.428280
THA.R	3.411034	MFG.R	3.377738	PHG.R	3.330617
MFG.R	3.403577	THA.R	3.364417	THA.R	3.321768
PHG.R	3.321308	PHG.R	3.341177	MFG.R	3.281550
PAL.L	3.276850	OLF.L	3.302985	OLF.L	3.253501
OLF.	3.229348	PAL.L	3.280203	PAL.L	3.227911
PAL.R	3.152530	PAL.R	3.184876	ROL.L	3.137098
ROL.L	3.141455	ROL.L	3.172325	PAL.R	3.122493
MFG.L	3.132499	SFGdor.R	3.118552	PHG.L	3.067575
SFGdor.R	3.132396	PHG.L	3.093056	SFGdor.R	3.034964
ITG.R	3.072447	MFG.L	3.089898	MFG.L	3.024577
PHG.L	3.066135	ITG.R	3.079373	ITG.R	3.010849
ACG.R	2.988173	ACG.R	2.996327	DCG.L	2.911139
DCG.L	2.949836	DCG.L	2.953595	ACG.R	2.907979
IFGtriang.L	2.917172	ROL.R	2.927337	ROL.R	2.901786
ROL.R	2.903213	IFGtriang.L	2.907755	ITG.L	2.846268
ITG.L	2.896850	ITG.L	2.893186	IFGtriang.L	2.843964
CAU.R	2.870713	TPOsup.L	2.848821	TPOsup.L	2.816354
LING.L	2.865661	ORBinf.R	2.834225	SMG.R	2.815568
STG.R	2.842634	SMG.R	2.830156	STG.R	2.806813
TPOsup.L	2.828565	LING.L	2.829542	LING.L	2.791497
SMG.R	2.822883	TPOmid.R	2.825690	SMA.R	2.774392
SMA.R	2.810670	STG.R	2.824868	TPOmid.R	2.772942
ORBinf.R	2.803069	SMA.R	2.816067	ORBinf.R	2.764503
TPOmid.R	2.791125	ORBinf.L	2.788808	ORBinf.L	2.733981
IFGoperc.L	2.769341	IFGoperc.L	2.756315	IFGoperc.L	2.712591
ORBinf.L	2.765282	TPOmid.L	2.730010	CAU.R	2.696260
SMA.L	2.721249	SMA.L	2.720204	SMA.L	2.679355
TPOmid.L	2.664277	CAU.R	2.697689	TPOmid.L	2.676023
MTG.R	2.661459	MTG.R	2.660613	HES.L	2.632641
HES.L	2.657243	HES.L	2.645314	MTG.R	2.628016
PreCG.R	2.582711	OLF.R	2.576091	OLF.R	2.578304
PCUN.R	2.578733	PreCG.R	2.573333	PreCG.R	2.547653
OLF.R	2.554337	PCUN.R	2.568711	PCUN.R	2.546613
TPOsup.R	2.547881	TPOsup.R	2.542616	TPOsup.R	2.532827
ORBmid.R	2.533984	ORBmid.R	2.523837	HES.R	2.489509
ORBsup.L	2.507120	ORBsup.L	2.507146	ORBmid.R	2.481680
HES.R	2.504298	HES.R	2.493194	ORBsup.L	2.451838
IOG.L	2.485049	ACG.L	2.464828	ACG.L	2.438174
ACG.L	2.464717	IOG.L	2.464171	IOG.	2.429005
ORBmid.L	2.442114	ORBmid.L	2.432890	STG.L	2.405026
STG.L	2.418517	STG.L	2.407583	ORBmid.L	2.393805
ORBsupmed.R	2.406942	ORBsupmed.R	2.402681	REC.R	2.373785
ORBsup.R	2.406147	MOG.R	2.399567	MOG.R	2.372705
MOG.R	2.397076	REC.R	2.399089	ORBsup.R	2.370415
REC.R	2.385842	ORBsup.R	2.394638	ORBsupmed.R	2.368406
LING.R	2.379107	REC.L	2.368514	SMG.L	2.358660
SMG.L	2.371757	SMG.L	2.368135	PCUN.L	2.357305
PCUN.L	2.371287	PCUN.L	2.365074	LING.R	2.338861
REC.L	2.347060	LING.R	2.364162	REC.L	2.334753
IOG.R	2.335631	IOG.R	2.316171	IOG.R	2.304650
PCG.R	2.276471	PCG.R	2.275727	PCG.R	2.277579
PCL.R	2.271374	PCL.R	2.265599	PCL.R	2.248829
MOG.L	2.256675	SFGmed.R	2.260130	PoCG.R	2.244600
SFGmed.R	2.256602	MOG.L	2.257062	SFGmed.R	2.235450
PoCG.R	2.255836	MTG.L	2.252054	MTG.L	2.234672
MTG.L	2.249654	PoCG.R	2.246999	MOG.L	2.231914
SFGdor.L	2.248007	SFGdor.L	2.236117	SFGdor.L	2.221235
ANG.R	2.236226	ANG.R	2.227490	ANG.R	2.217417
SOG.L	2.187115	SOG.L	2.183815	SOG.L	2.172867
SPG.L	2.167275	SPG.L	2.159774	SPG.GL	2.155164
PCG.L	2.134401	PCG.L	2.140541	PCG.L	2.137398
ORBsupmed.L	2.125971	ORBsupmed.L	2.125862	ORBsupmed.L	2.111867
CAL.R	2.119823	CAL.R	2.115090	ANG.L	2.109015
ANG.L	2.119235	ANG.L	2.113897	PoCG.L	2.108075
PoCG.L	2.118919	PreCG.L	2.111897	CAL.R	2.107400
PreCG.L	2.116344	SFGmed.L	2.111101	SPG.R	2.105448
SPG.R	2.108842	PoCG.L	2.110284	PreCG.L	2.105135
SFGmed.L	2.101027	SPG.R	2.107186	SFGmed.L	2.093920
IPL.L	2.098824	IPL.L	2.097696	IPL.L	2.093085
SOG.R	2.073710	SOG.R	2.075040	IPL.R	2.071129
IPL.R	2.071750	IPL.R	2.073850	SOG.R	2.069683
CUN.R	2.069898	CUN.R	2.066857	CUN.R	2.064225
CAL.L	2.058440	CAL.L	2.054236	CAL.L	2.050401
PCL.L	2.044241	PCL.L	2.043656	PCL.L	2.046279
CUN.L	2.000000	CUN.L	2.000000	CUN.L	2.000000
